# Disulfidptosis-related lncRNAs signature predicting prognosis and immunotherapy effect in lung adenocarcinoma

**DOI:** 10.18632/aging.205911

**Published:** 2024-06-10

**Authors:** Suifeng Hong, Yu Zhang, Dongfeng Wang, Huaying Wang, Huihui Zhang, Jing Jiang, Liping Chen

**Affiliations:** 1 Department of Respiratory and Critical Care Medicine, The Affiliated People's Hospital of Ningbo University, Ningbo 315400, China; 2 Department of Oncology Radiation, Shuguang Hospital Affiliated to Shanghai University of Traditional Chinese Medicine, Shanghai 200433, China; 3 Dongying People's Hospital (Dongying Hospital of Shandong Provincial Hospital Group), Dongying, Shandong 257091, China

**Keywords:** disulfidptosis, lung adenocarcinoma, long non-coding RNAs, prognosis model, drug sensitivity

## Abstract

Purpose: Lung adenocarcinoma (LUAD) is a prevalent malignant tumor worldwide, with high incidence and mortality rates. However, there is still a lack of specific and sensitive biomarkers for its early diagnosis and targeted treatment. Disulfidptosis is a newly identified mode of cell death that is characteristic of disulfide stress. Therefore, exploring the correlation between disulfidptosis-related long non-coding RNAs (DRGs-lncRNAs) and patient prognosis can provide new molecular targets for LUAD patients.

Methods: The study analysed the transcriptome data and clinical data of LUAD patients in The Cancer Genome Atlas (TCGA) database, gene co-expression, and univariate Cox regression methods were used to screen for DRGs-lncRNAs related to prognosis. The risk score model of lncRNA was established by univariate and multivariate Cox regression models. TIMER, CIBERSORT, CIBERSORT-ABS, and other methods were used to analyze immune infiltration and further evaluate immune function analysis, immune checkpoints, and drug sensitivity. Real-time polymerase chain reaction (RT-PCR) was performed to detect the expression of DRGs-lncRNAs in LUAD cell lines.

Results: A total of 108 lncRNAs significantly associated with disulfidptosis were identified. A prognostic model was constructed by screening 10 lncRNAs with independent prognostic significance through single-factor Cox regression analysis, LASSO regression analysis, and multiple-factor Cox regression analysis. Survival analysis of patients through the prognostic model showed that there were obvious survival differences between the high- and low-risk groups. The risk score of the prognostic model can be used as an independent prognostic factor independent of other clinical traits, and the risk score increases with stage. Further analysis showed that the prognostic model was also different from tumor immune cell infiltration, immune function, and immune checkpoint genes in the high- and low-risk groups. Chemotherapy drug susceptibility analysis showed that high-risk patients were more sensitive to Paclitaxel, 5-Fluorouracil, Gefitinib, Docetaxel, Cytarabine, and Cisplatin. Additionally, RT-PCR analysis demonstrated differential expression of DRGs-lncRNAs between LUAD cell lines and the human bronchial epithelial cell line.

Conclusions: The prognostic model of DRGs-lncRNAs constructed in this study has certain accuracy and reliability in predicting the survival prognosis of LUAD patients, and provides clues for the interaction between disulfidptosis and LUAD immunotherapy.

## INTRODUCTION

Lung cancer is the second most common form of malignant tumour, with the highest mortality rate among all malignant tumours [1, 2]. According to 2021 statistics, lung cancer accounts for approximately 22% of cancer-related deaths, with a five-year survival rate of only 21% [3]. In recent years, medical technologies such as surgery, chemotherapy, radiotherapy, and targeted therapy have advanced in the treatment of lung cancer [4]. However, early-stage lung cancer, especially peripheral lung adenocarcinoma (LUAD) with imaging features of ground-glass nodules, is difficult to detect early due to its lack of obvious symptoms. Therefore, most LUAD patients have already reached the intermediate or late stage at the time of diagnosis, losing the best opportunity for surgery [5]. For this group of people, the primary treatment methods are chemotherapy, radiotherapy, molecular targeted therapy, and immunotherapy [6]. However, despite the diverse treatments available, there has been no significant improvement in the overall survival rate of LUAD to date. These treatment methods have many shortcomings, including high side effects and poor tolerance in relapsed patients [7]. Therefore, the development of new early diagnostic methods, more sensitive biomarkers, and treatment targets is urgently needed for LUAD.

In 2023, Professor Gan Boyi’s team reported a new mode of cell death called ‘disulfidptosis’. This form of cell death is induced by a shortage of NADPH under glucose starvation [8]. The shortage leads to the overexpression of the cystine transporter solute carrier family 7 member 11 (SLC7A11) in cancer cells, which induces abnormal accumulation of disulfide (such as Cystine, etc.) in cells, ultimately leading to cell death. In this research mechanism, the process of cell reduction of cystine to cysteine is blocked due to an insufficient supply of NADPH caused by glucose starvation. This blockage leads to the production of a large number of disulfide bonds between actin molecules, inducing disulfide stress. The stress activates the Rac/WAVE regulatory complex-7 subunit of actin-related protein 2/3 (Arp2/3) signal pathway, causing disarray of the cell skeleton and ultimately inducing disulfidptosis. Currently, research on disulfidptosis is focused on cancer cell lines that overexpress SLC7A11 and are under glucose starvation [9, 10]. However, the role and mechanism of disulfidptosis in LUAD are still unclear.

Long non-coding RNA (lncRNA) is defined as RNA longer than 200 nucleotides. Although lncRNA is not involved in protein translation, it plays a very important role in gene regulation. LncRNA can affect the translation and stability of cytoplasmic mRNA and influence signal transduction pathways by regulating the function of chromatin and the function of membrane-free nucleosomes, thereby affecting gene expression in various biological physiological, and pathological environments [11, 12]. In particular, abnormal lncRNA expression may be associated with multiple biological events, such as ferroptosis [13]. LINC00336 reduces ferroptosis in lung cancer through competitive endogenous RNA activity [14]. LncRNA P53RRA induces tumor suppression by isolation of nuclear p53 and promotes ferroptosis and apoptosis [15]. As a newly discovered mode of cell death, disulfidptosis has different causes than programmed cell death such as apoptosis [16], ferroptosis [17], and pyroptosis [18]. Therefore, identifying disulfidptosis-related lncRNAs associated with LUAD prognosis is critical to developing accurate prognostic assessment and treatment.

In this study, we established and validated a highly predictive disulfidptosis-related prognostic model for LUAD. By constructing risk scores, we analyzed the clinical value in predicting clinical prognosis, immune induction, tumor mutation compliance, and immunotherapy response. Our study may provide a promising tool for predicting the prognosis of LUAD patients and offer new theoretical references for elucidating the molecular mechanisms of LUAD.

## METHODS

### Data refinement and preprocessing

RNA-seq data and patient clinical information data, including patients with overall survival (OS) time and status (59 normal samples and 541 tumor samples), were extracted from The Cancer Genome Atlas (TCGA) database (https://portal.gdc.cancer.gov/). Copy number variation (CNV) data and somatic mutations were downloaded from TCGA. The prognostic value of 16 disulfidptosis-related genes (DRGs) was assessed using the Kaplan-Meier (K-M) method. The 16 DRGs were obtained from previous studies (GYS1, NDUFS1, OXSM, LRPPRC, NDUFA11, NUBPL, NCKAP1, RPN1, SLC3A2, SLC7A11, ACTB, FLNB, MYH9, PRDX1, TLN1, FLNA) [8].

### Detection of the differentially expressed genes (DEGs) and disulfidptosis-related lncRNAs (DRGs-lncRNAs)

After extracting mRNA and lncRNA expression data from the TCGA database, Pearson correlation coefficients were utilized to perform correlation analysis between the expression levels of lncRNAs and DRGs. By determining the relationship between lncRNAs and DRGs (correlation coefficient >0.40, *P* < 0.001), DRGs-lncRNAs were identified. In addition, we found differentially expressed DRGs-lncRNAs in 59 normal tissues and 541 LUAD tissues using the R package “limma” package [19], with a defined criterion of *P* < 0.05 and |log2 (fold change)| >1. Meanwhile, differential expression analysis was also performed on DRGs, with a significantly differential expression threshold set at *P* < 0.05 and |log2 (fold change)| >1. We used the R software package “ggplot2” to visualize differentially expressed DRGs-lncRNAs and genes.

### Construction of the disulfidptosis gene signature

We determined the prognostic variables of LAUD and established its prognostic features by the least absolute shrinkage and selection operator (LASSO) Cox regression analysis. Patients were divided into the high group and the low group according to the median value of the DRGs scores. The two groups’ overall survival (OS) and progression-free survival (PFS) were determined using Kaplan-Meier curves with log-rank tests. Time-dependent ROC analysis was used to estimate the performance of the model.

### The predictive power of DRGs-lncRNAs models

To thoroughly examine the predictive performance of the DRGs-lncRNAs model, the Receiver Operating Characteristic (ROC) analysis was performed on the training group, the test group, and the entire cohort using the “timeROC” package. Independence was determined by univariate and multivariate Cox regression analyses of the entire cohort using a DRGs-lncRNAs model and corresponding clinicopathological data.

### Correlation enrichment analysis

We employed the R package “clusterProfiler” [20] to perform Gene Ontology (GO) and Kyoto Encyclopedia of Genes and Genomes (KEGG) analyses. The “limma” package was used to identify the DEGs between molecular clusters based on DRG [19]. DEGs with an adjusted *P*-value < 0.01 and |logFC [fold change] | >1 were considered significant. The “GSVA” package was used to identify DRG-related differences in biological function in the MsigDB database.

### Tumor mutational burden, drug sensitivity analysis, and immune cell infiltration

Download tumor mutational burden (TMB) data (MAF format) from the TCGA database and analyze it in high-risk and low-risk groups across the cohort using the “maftools” package [21], and calculate TMB (mutations per million bases) for each patient and visualize the mutation data using a waterfall plot.

We assessed tumor-infiltrating immune cells by EPIC algorithms, CIBERSORT-ABS, MCP-counter, XCELL, QUANTISEQ, CIBERSORT, and TIMER. We also compared immune checkpoints and drug sensitivity from the high-risk and low-risk groups. Furthermore, we quantified the differences in TME scores and immune-related gene expression between the two risk groups.

### Cell lines and culture

Human LUAD cell lines (A549 and PC9) and the human bronchial epithelial (HBE) cell line were obtained from the Institute of Biochemistry and Cell Biology of the Chinese Academy of Sciences (Shanghai, China). They were maintained in Roswell Park Memorial Institute (RPMI) 1640 supplemented with 10% heat-inactivated fetal bovine serum (FBS), 100 U/mL of penicillin, and 100 μg/mL streptomycin sulfate. Cultures were incubated in a humidified atmosphere containing 5% CO_2_ at 37°C.

### RNA preparation and quantitative real-time PCR

Total RNA was extracted from tissues or cultured cells using TRIzol reagent (Invitrogen, Carlsbad, CA, USA). For qRT-PCR, 1 μg RNA was reverse transcribed into cDNA with a Reverse Transcription Kit (Takara, Dalian, China). Real-time PCR was performed with SYBR Premix ExTaq II Kit (Takara, Dalian China). Data were normalized to GAPDH levels. The sequence of primers used in the detection is shown in Supplementary Table 1. The qRT-PCR assays and data collection were performed on ABI 7500, and relative expression was assessed by the 2^−ΔΔCt^ method, and converted to fold changes using the 2^−ΔΔCt^ method.

### Statistical analysis

R version 4.3.0 was used for statistical analysis. The log-rank test was used for survival analysis. The Student’s *t*-test was utilized to test normally distributed groups, while the Wilcoxon test was used to test non-normally distributed variables. Spearman analysis was used for correlation analysis. For the analysis of clinical features, Chi-square tests or Fisher’s exact tests were utilized. A *P*-value less than 0.05 was considered statistically significant.

### Data availability statement

The original contributions presented in the study were included in the article/Supplementary Material. Further inquiries can be directed to the corresponding author.

## RESULTS

### Expression of DRG-lncRNAs in LUAD

**Figure 1:**
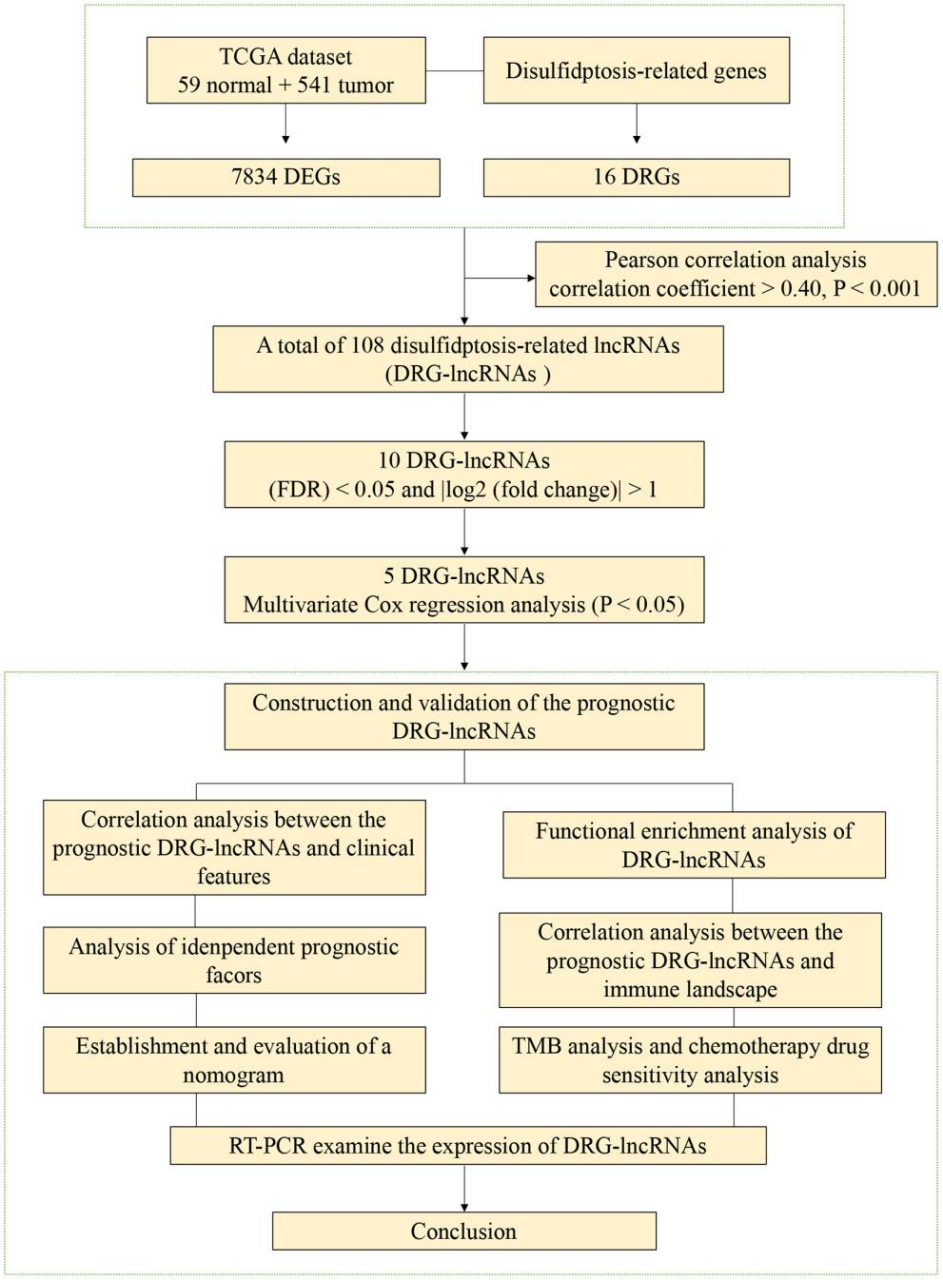
The flowchart of this study.

**Figure 2:**
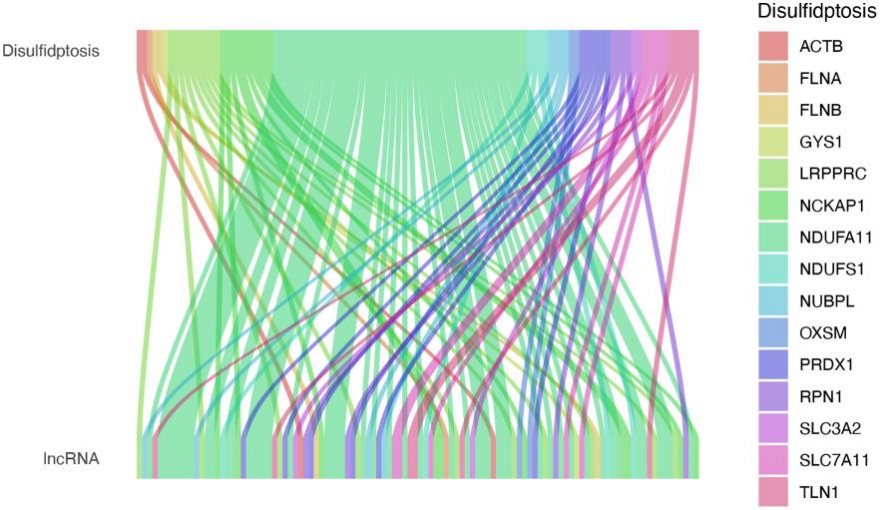
**Expression of DRG-lncRNAs in LUAD.** DRG-lncRNAs, disulfidptosis-related long non-coding RNAs. Abbreviation: LUAD; lung adenocarcinoma.

The flowchart of this study is shown in Figure 1. We analyzed the expression levels of 16 DRGs in the lung tissues of 541 LUAD patients and 59 healthy individuals downloaded from the TCGA-LUAD dataset. Subsequently, co-expression analysis was performed to identify DRG-lncRNAs (Figure 2), and a total of 108 lncRNAs significantly associated with disulfidptosis were identified (Supplementary Table 2).

### Construction of the disulfidptosis-related prognostic signature

**Figure 3:**
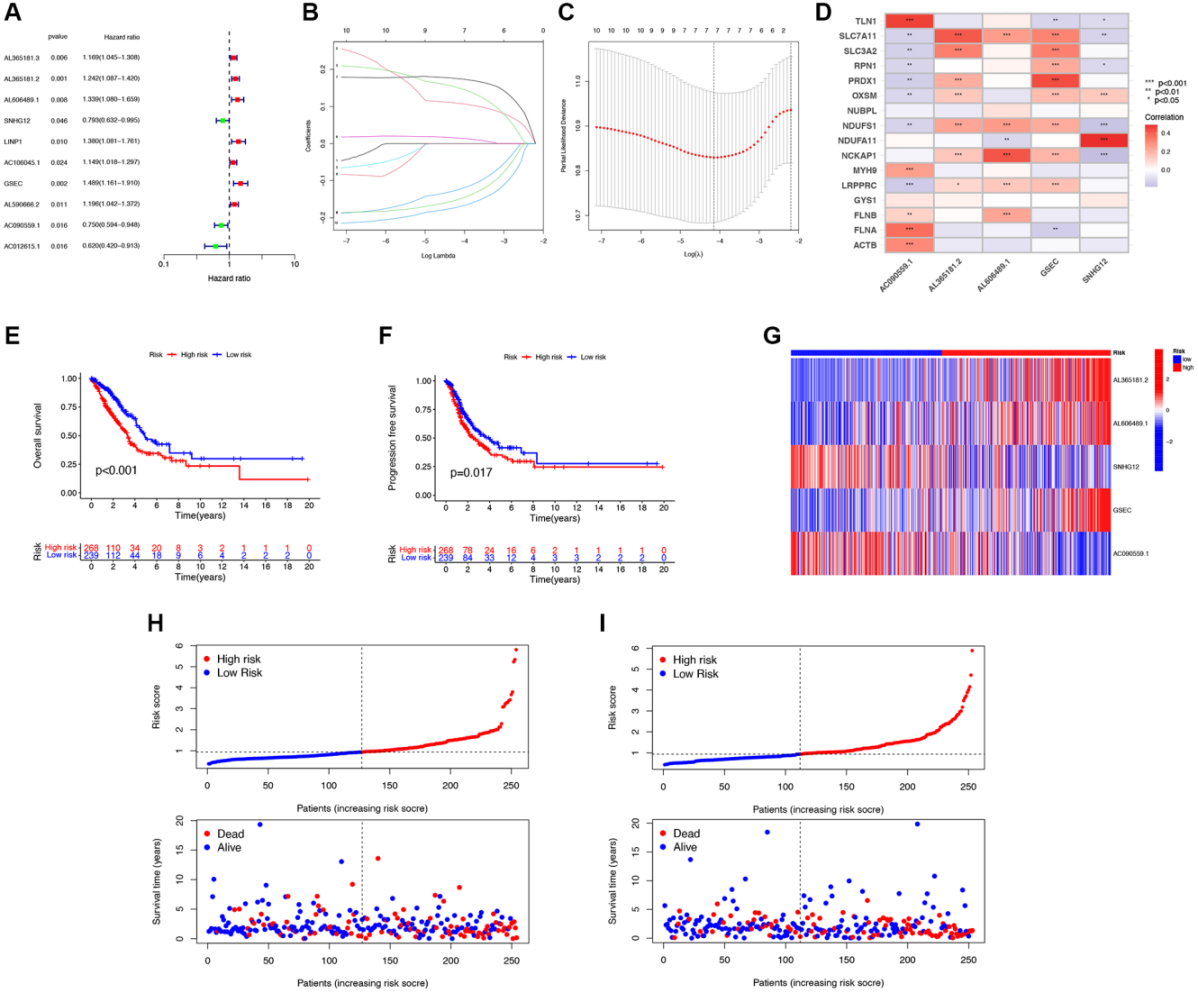
**Construction of the disulfidptosis-related prognostic signature.** (**A**) 10 DRG-lncRNAs significantly correlated with the survival prognosis of LUAD patients. (**B**) LASSO regression based on optimal parameter (lambda) construction model. (**C**) LASSO regression coefficient curve. (**D**) Multivariate Cox regression analysis of DRG-lncRNA with prognostic significance. (**E**, **F**) The Kaplan-Meier curve shows different OS and PFS between the low-risk and high-risk groups. (**G**) A heatmap shows the differential expression of DRG-lncRNAs in the high-risk and low-risk groups. (**H**) The risk curve of the training group is reordered by disulfidptosis related signature and the scatter plot of the sample survival overview. The green and red dots represent survival and death, respectively. (**I**) The risk curve of the test group is reordered by disulfidptosis related signature and the scatter plot of the sample survival overview. The green and red dots represent survival and death, respectively. Abbreviations: DRG-lncRNAs: disulfidptosis-related long non-coding RNAs; LUAD: lung adenocarcinoma; LASSO: least absolute shrinkage and selection operator; OS: overall survival; PFS: progression-free survival.

Patients in the entire cohort (*n* = 541) were randomly divided into the training group (*n* = 406) and the test group (*n* = 135) in a 3:1 ratio. Through univariate Cox regression analysis, 10 DRG-lncRNAs significantly correlated with the survival prognosis of LUAD patients were obtained (*P* < 0.05, Figure 3A). LASSO regression was applied to the 7 DRG-lncRNAs (Figure 3B, 3C).

Multivariate Cox regression analysis was performed on 10 DRG-lncRNAs with prognostic significance, and a prognostic model consisting of 5 DRG-lncRNAs was further screened out, including AL365181.2, AL606489.1, SNHG12, GSEC, and AC090559.1 (Figure 3D). All of these lncRNAs were identified as risk factors for prognosis in LUAD patients (Hazard Ratio >1). The risk score formula was used to calculate the risk score for LUAD patients, and they were divided into high-risk and low-risk groups according to the median risk score.

According to the median risk score, there was a significant difference in the OS among LUAD patients (*P* < 0.05), confirming that the OS of the low-risk group was significantly higher than that of the high-risk group (Figure 3E). Compared with the low-risk group, the PFS of LUAD patients in the high-risk group was significantly reduced (Figure 3F). A heatmap was drawn based on the differential expression of DRG-lncRNAs in the high-risk and low-risk groups (Figure 3G). Scatter plots and risk curves were used to display the survival status and risk score of LUAD patients (Figure 3H, 3I). The mortality rate and hazard ratio were higher in the high-risk group than in the low-risk group.

### Clinical features and evaluation of the prognostic ability

**Figure 4:**
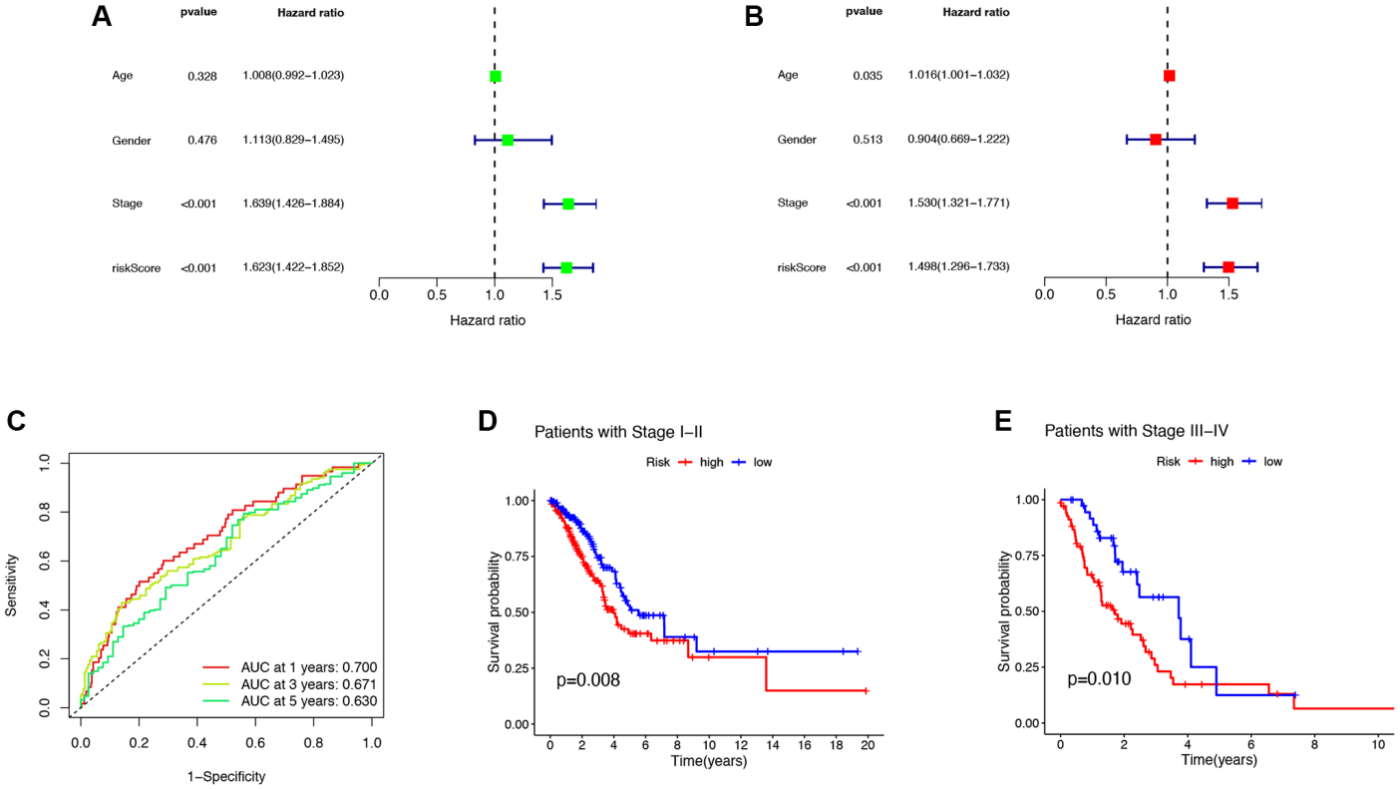
**Clinical features and evaluation of the prognostic ability.** (**A**, **B**) The univariate and multivariate Cox regression analysis of risk scores, age, gender, grade, stage. (**C**) One- three- and five-year AUC in the risk score. (**D**, **E**) Clinical prognosis analysis of LUAD patients with low- and high scores among stage. Abbreviations: AUC: area under curve; LUAD: lung adenocarcinoma.

The forest plot results obtained by univariate and multivariate Cox regression analysis showed that both Stage and risk scores were independent prognostic factors in LUAD patients (*P* < 0.05, Figure 4A, 4B). To evaluate the accuracy of the risk score and clinical characteristics in predicting the prognosis of LUAD patients, the ROC curve results showed that the AUC of the risk score for 1 year and 3 years were 0.700 and 0.630, respectively (Figure 4C). The K-M curve results showed that LUAD patients with the same pathological grading had shorter survival times and worse prognoses with higher risk scores (*P* < 0.05, Figure 4D, 4E). This indicated that the model had certain reliability and accuracy in predicting the prognosis of LUAD patients.

### Functional enrichment analysis of DRG-lncRNAs

**Figure 5:**
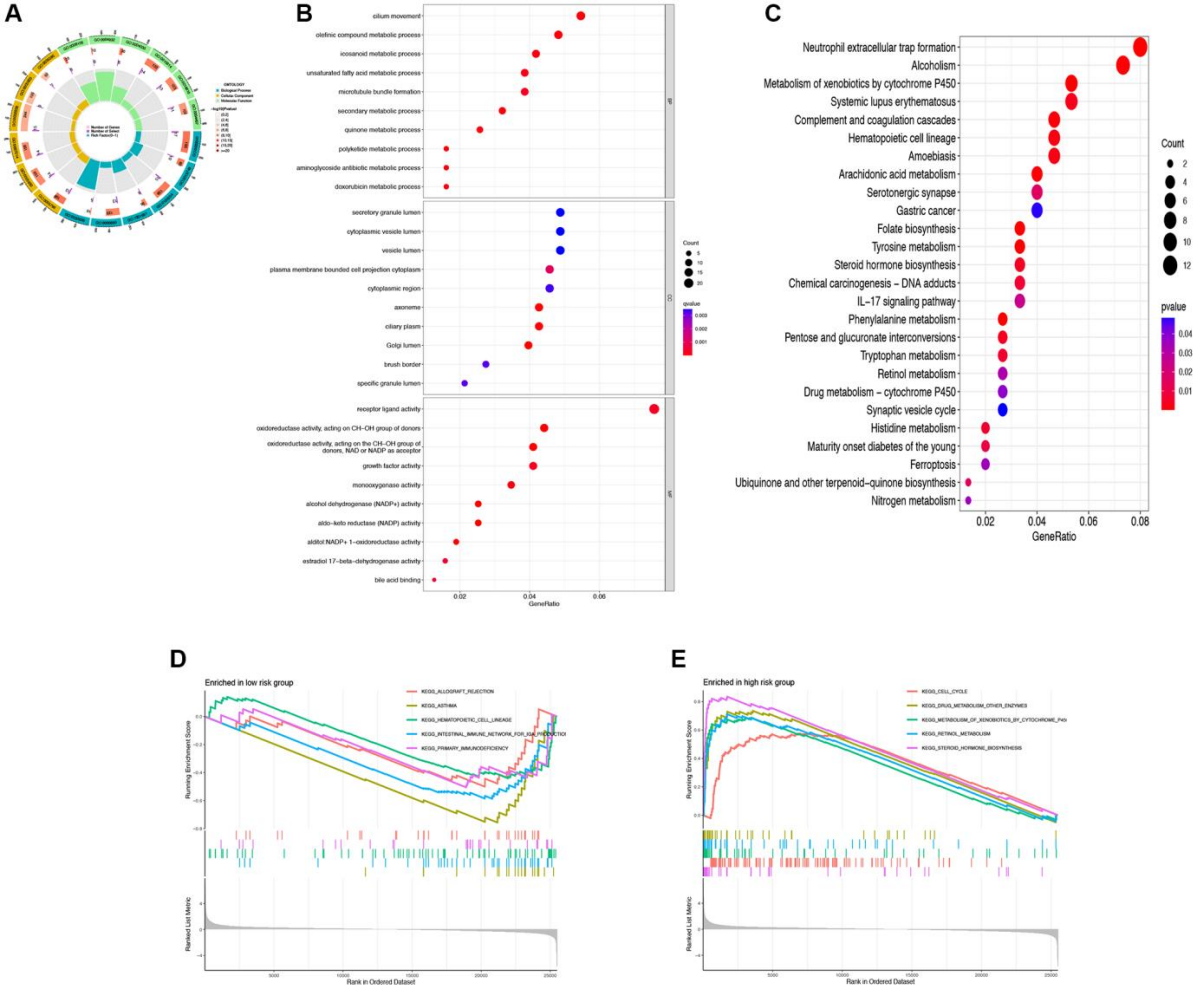
**Functional enrichment analysis of DRG-lncRNAs.** (**A**–**C**) GO and KEGG enrichment analysis of DRG-lncRNAs. (**D**) Pathways of enrichment of highly and lowly expressed genes in the high-risk group. (**E**) Pathways of enrichment of highly and lowly expressed genes in the low-risk group. Abbreviations: DRG-lncRNAs: disulfidptosis-related long non-coding RNAs; GO: Gene Ontology; KEGG: Kyoto Encyclopedia of Genes and Genomes.

To investigate the mechanisms underlying the impact of DRG-lncRNAs characteristics on LUAD prognosis, this study conducted GO and KEGG enrichment analyses on lncRNAs based on high/low-risk scores. GO analysis showed that the cilium movement, secretory granule lumen, and receptor-ligand activity were the richest terms in biological processes (BP), cellular components (CC), and molecular functions (MF) (Figure 5A, 5B). The KEGG enrichment analysis showed that multiple pathways were associated with DRG-lncRNAs features (Figure 5C). The GSEA subsequently uncovered that the low-risk group was enriched in asthma and the high-risk group was enriched in steroid hormone biosynthesis (Figure 5D, 5E).

### The correlation between DRG-lncRNAs and immune cell infiltration

**Figure 6:**
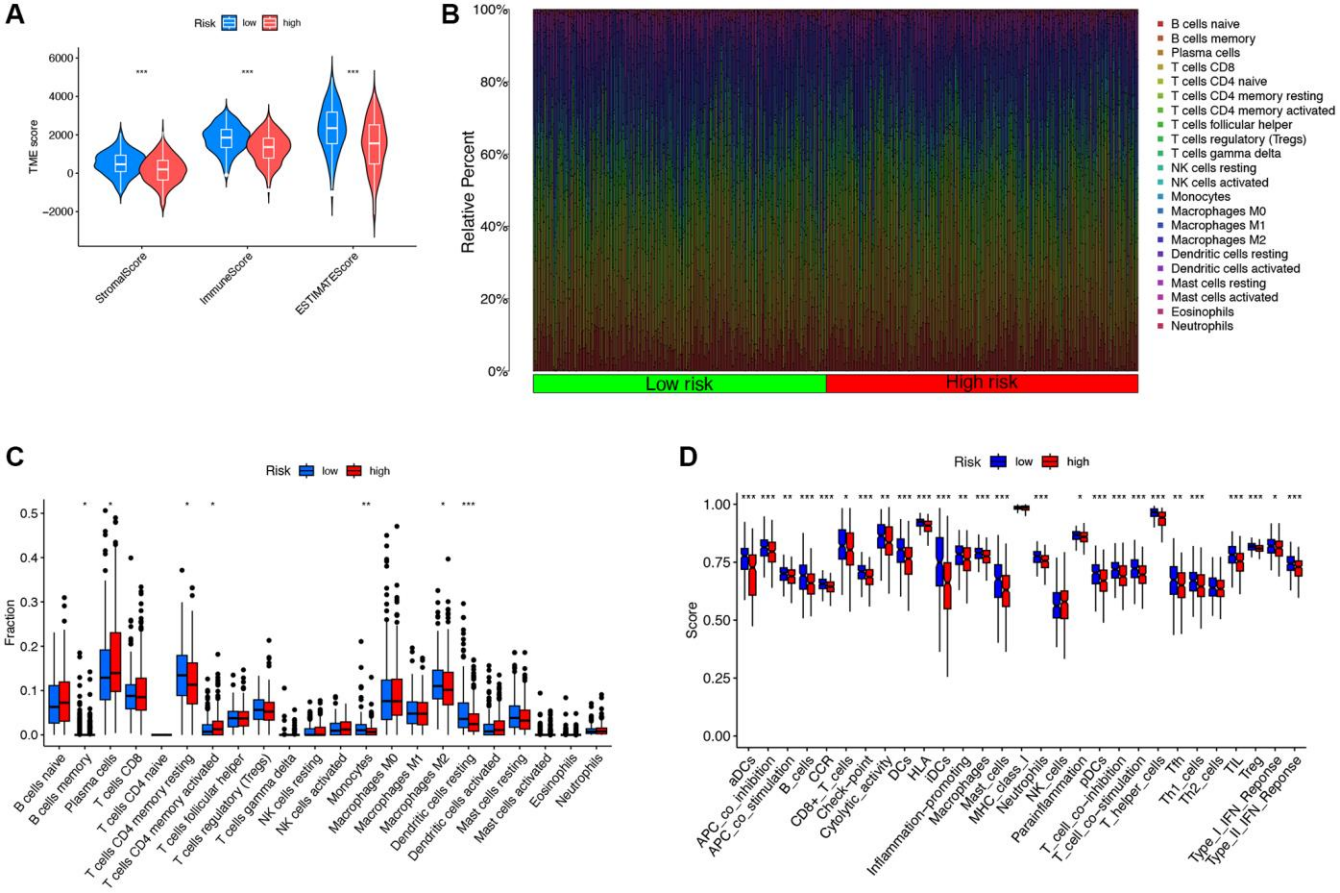
**The correlation between DRG-lncRNAs and immune cell infiltration.** (**A**) Results of differences in stromal cell score, immune cell score, and comprehensive score among LUAD patients under different risk groups. (**B**) Immune response heatmaps for high-risk and low-risk groups based on CIBERSORT, CIBERSORT ABS, XCELL, MCPcounter, QUANTISEQ, EPIC, and TIMER algorithms. (**C**) Abundances of infiltrating immune cells between high-risk and low-risk groups. (**D**) Differential expression of immune function scores between high-risk and low-risk groups. Abbreviations: DRG-lncRNAs: disulfidptosis-related long non-coding RNAs; LUAD: lung adenocarcinoma; TME: tumor microenvironment.

The results of TME scoring showed that there was a significant difference in the stromal cell score, the immune cell score, and the comprehensive score between the high-risk and low-risk groups (Figure 6A). The immune response heatmaps based on seven algorithms, CIBERSORT, CIBERSORT ABS, XCELL, MCPcounter, QUANT ISEQ, EPIC, and TIMER, were shown in Figure 6B. To confirm the role of DRG-lncRNAs in regulating LUAD immune cell infiltration, we analyzed the abundance difference of infiltrating immune cells in high/low-risk groups (Figure 6C). The results of the immune function enrichment analysis showed that there was no significant difference in MHC class I, NK cells, and Th2 cells among the 22 types of immune cells, while the rest were significantly significant in both high-risk and low-risk groups (Figure 6D).

### TMB analysis and survival analysis of TMB

**Figure 7:**
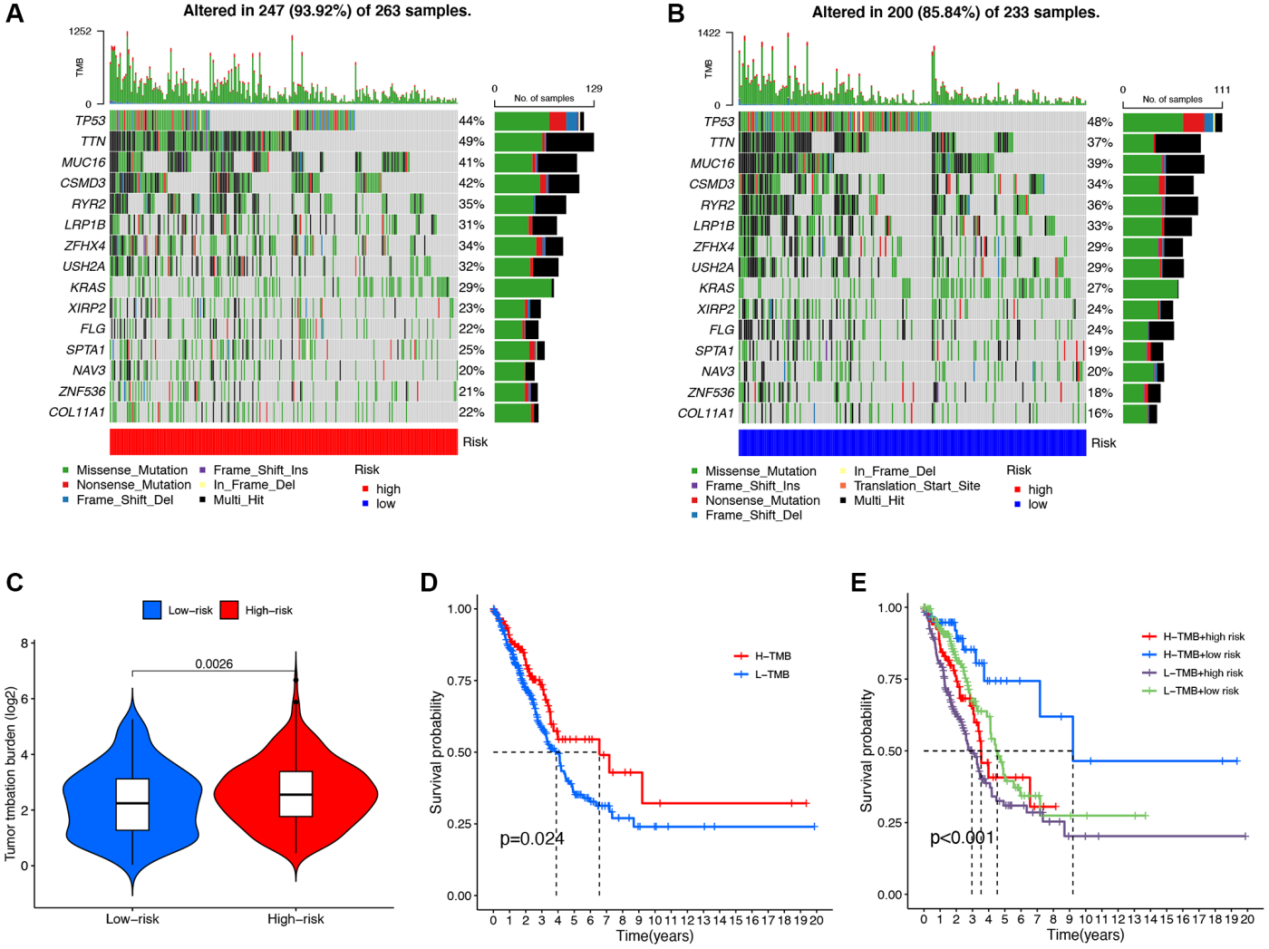
**Mutation analysis of the DRG-lncRNAs based on the risk score model.** (**A**, **B**) The waterfall diagram shows the genes that most frequently undergo somatic mutations under different risk groups. (**C**) The difference in tumor mutation burden between high- and low-risk score groups. (**D**) Kaplan-Meier curves of high and low TMB groups. (**E**) Kaplan-Meier curves of four groups classified by risk score and TMB. Abbreviations: DRG-lncRNAs: disulfidptosis-related long non-coding RNAs; TMB: tumor mutational burden; H: high; L: low.

We analyzed the mutation data of the TCGA dataset and displayed the mutation information of each gene in the sample through a waterfall plot. We compared the top 20 mutated genes in the high-risk group (Figure 7A) and the low-risk group (Figure 7B), among which TP53 (44% vs. 48%), TTN (49% vs. 37%), MUC16 (41% vs. 39%), and CSMD3 (42% vs. 34%) were the most common mutated genes. Compared with the low-risk group, the frequency of mutations was higher in the high-risk group (93.92% vs. 85.84%). In addition, there was a significant increase in TMB levels in the high-risk group compared to the low-risk group (*P* = 0.0026, Figure 7C). The Kaplan-Meier survival analysis demonstrated a significant increase in OS among LUAD patients in the high TMB group compared to the low TMB group (*P* = 0.0026, Figure 7D). In the high TMB and low-risk group, the OS of LUAD patients significantly increased (Figure 7E).

### DRG-lncRNAs correlate with chemotherapy drug sensitivity

**Figure 8:**
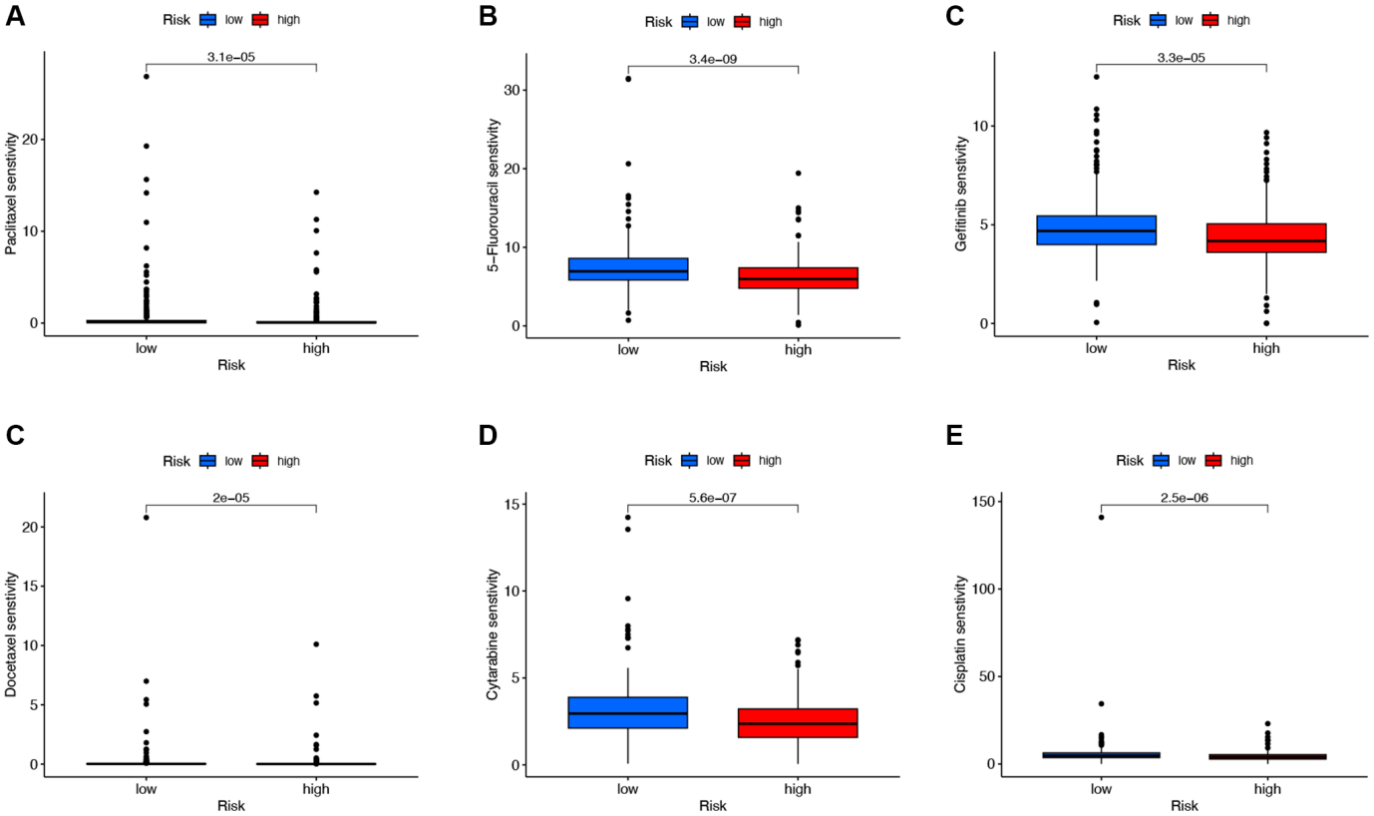
**Analysis of chemotherapeutic drugs sensitivity.** (**A**–**E**) Chemotherapeutic drugs sensitivity analysis of Paclitaxel, 5-Fluorouracil, Gefitinib, Docetaxel, Cytarabine, and Cisplatin in the low-risk and high-risk groups.

To explore chemotherapeutic drugs that may be sensitive to LUAD patients, this study used the R software package based on the GDSC2 database to determine the IC_50_ of chemotherapy drugs and performed drug sensitivity analysis (Figure 8). The results showed that compared with the low-risk group, the IC_50_ values of Paclitaxel, 5-Fluorouracil, Gefitinib, Docetaxel, Cytarabine, and Cisplatin were significantly reduced in the high-risk group. These results indicate that Paclitaxel, 5-Fluorouracil, Gefitinib, Docetaxel, Cytarabine, and Cisplatin may be sensitive chemotherapeutic drugs for treating LUAD patients with high risk.

### Exploring the expression pattern of the identified DRG-lncRNAs in the risk model

**Figure 9:**
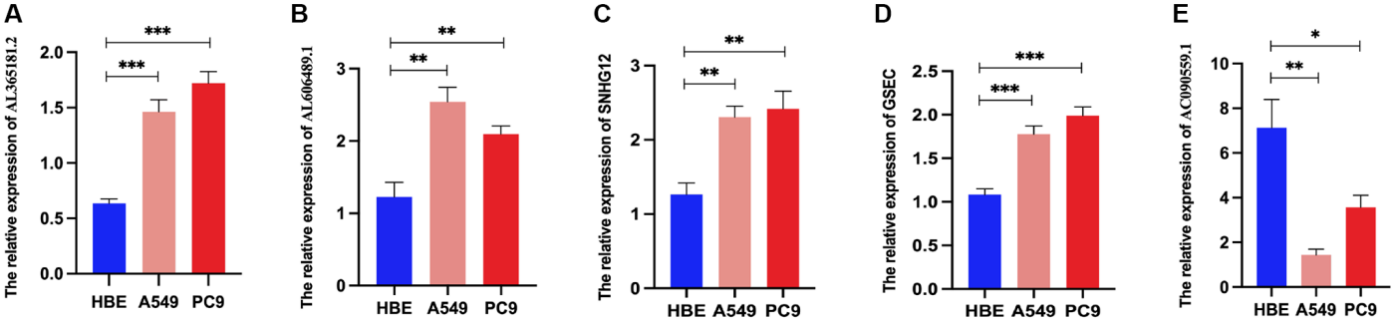
**Relative DRG-lncRNAs expression levels in LUAD cell lines.** (**A**–**E**) The mRNA expression level of AL365181.2, AL606489.1, SNHG12, GSEC, and AC090559.1 in LUAD cell lines (A549 and PC9) and the human bronchial epithelial cell line (HBE). Abbreviations: DRG-lncRNAs: disulfidptosis-related long non-coding RNAs; LUAD: lung adenocarcinoma. ^**^P < 0.01, ^***^P < 0.001.

Then, we examined the expression of DRG-lncRNAs in a panel of LUAD cell lines (A549 and PC9) and the human bronchial epithelial cell line (HBE) by RT-PCR. Compared with the HBE, the expression of AL365181.2, AL606489.1, SNHG12, and GSEC was increased in LUAD cell lines (Figure 9A–9D), and AC090559.1 was decreased in LUAD cell lines (Figure 9E).

## DISCUSSION

With the continuous deepening of tumor research, LUAD has made positive progress in detection, diagnosis, and treatment. However, this disease remains one of the most deadly malignancies due to the complexity of its molecular and genetic processes [22]. As an emerging biomarker, lncRNAs play an important role in the occurrence and development of different tumors, including LUAD [23]. In addition, many lncRNAs participate in the occurrence, development, and drug resistance of malignant tumors and have been identified as novel biomarkers and therapeutic targets for tumor diagnosis and treatment [24–26]. Nevertheless, it is still uncertain whether DRG-lncRNAs can predict LUAD patients’ prognosis. Therefore, we developed a prediction model for LUAD and improved patient survival rates by screening for DRG-lncRNAs.

To prevent overfitting, we used LASSO regression to reduce the dimensionality of the data. We identified 10 DRG-lncRNAs (AL365181.3, AL365181.2, AL606489.1, SNHG12, LINP1, AC106045.1, GSEC, AL590666.2, AC090559.1, and AC012615.1) that are closely associated with the OS of LUAD patients. Some of these DRG-lncRNAs have been previously reported and are closely linked to the occurrence and progression of tumors. Chen et al.’s research team found that LINP1 can increase pancreatic cancer metastasis through adsorbing microRNA-491-3p [27]. In addition, studies have shown that SNHG12 is upregulated in NSCLC tissues and cells, and facilitated immune escape through the HuR/PD-L1/USP8 axis [28]. In other LUAD-related prognostic models, AL365181.3 and AL365181.2 have been identified as indicators that can accurately predict prognosis, and their overexpression is considered a protective factor for the prognosis of LUAD patients [29, 30]. However, their specific mechanisms of action in LUAD are not yet fully clear and require further study. Research on the remaining DRG-lncRNAs is limited, and although little is known about them, their importance should not be underestimated.

In addition, we constructed a prognostic model that predicts the survival of LUAD patients based on 16 DRGs, which can be independently used as a prognostic indicator for LUAD. This model accurately divides patients into two groups: low-risk group and high-risk group. In the overall cohort, the low-risk group showed a better prognosis. In addition, we evaluated the effectiveness of this prognostic model in predicting prognosis by constructing ROC curves, and the results showed that the AUC values of the risk score for 1 year and 3 years were 0.700 and 0.630, respectively. To date, several evaluation methods have been used to assess the prognosis of patients with LUAD. In a study by Li et al., the investigators constructed a prognostic model of LUAD based on ferroptosis-related genes. In their study, the AUC value predicting 1-year survival was 0.698, which is lower than our prognostic model [31]. Therefore, the 10 DRG-lncRNAs model we studied could be a good prognostic model for LUAD.

One of the major challenges in immune therapy for LUAD patients is the lack of understanding of tumor heterogeneity, complexity, and immune evasion mechanisms. Additionally, specific biomarkers to evaluate the benefits of tumor immune therapy are insufficient. Therefore, the discovery of new immune targets and prognostic indicators is of great importance [32]. Several clinical-pathological studies have shown a correlation between gene mutations and immune therapy response [33, 34]. In this study, the high-risk group showed a significantly increased frequency of mutation events and TMB levels. This finding suggests that LUAD patients may benefit from tumor immune therapy, such as programmed cell death protein 1 (PD-1) inhibitors. Because patients with higher TMB levels have significantly improved response rates to immune checkpoint inhibitors compared with patients with lower TMB levels [35, 36]. In addition, studies have shown that the effectiveness of immune therapy depends on the coordinated responses of innate and acquired immune cells [37]. Furthermore, tumor-infiltrating immune cells may be a valuable prognostic tool in cancer treatment. In this study, we used seven algorithms such as CIBERSORT, CIBERSORT-ABS, XCELL, MCPcounter, QUANTISEQ, EPIC, and TIMER to study the potential of the 10 DRG-lncRNAs model to reflect the immune microenvironment status of LUAD and focused on using the CIBERSORT algorithm to detect the relative proportions of 22 different types of immune cells in tumor tissues in the TCGA collection. The results showed a significant increase in the number of activated CD4 memory T cells, resting NK cells, and resting macrophages in the high-risk group, confirming the role of the 10 DRG-lncRNAs model in regulating tumor immune cell infiltration. These findings suggest that the proposed model in this study can accurately predict the immune therapy efficacy of LUAD patients.

Metabolic reprogramming is one of the important features of cancer, which typically leads to increased uptake of nutrients crucial in biosynthesis and bioenergetics processes by cancer cells, such as glucose and amino acids including glutamine [38]. Cancer cells primarily achieve this through upregulating transporters for glucose and amino acid uptake. Accordingly, some cancer cells undergo cell death upon glucose or amino acid restriction while normal cells can survive under the same conditions. This nutrient dependency provides potential metabolic vulnerabilities for targeted therapy against cancer [39]. The research by Gan Boyi’s team indicates that elevated expression of SLC7A11 promotes metabolic vulnerability resulting in disulfidptosis, which may be an effective strategy for treating tumors.

With the introduction of the concepts of precision medicine and personalized therapy and the deepening of the application of machine learning in the medical field, researchers have also begun to try to apply these concepts and methods to the diagnosis, personalized medication and prognosis evaluation of cancer patients [40]. Patient clinical information, pathological information, imaging results, laboratory test results, epidemiological characteristics, genomic and proteomic data, and other data can be used as predictors for tumor prediction models [41]. The synthesis of many different data types for model construction is a trend in current research [42]. Machine learning methods have greatly accelerated the interpretation of medical big data, especially genetic and genomic data. Guyon et al. used a support vector machine (SVM) approach to construct a predictive model by selecting a subset of characteristic genes to distinguish between tumors and normal tissues [43]. Statnikov et al. systematically and comprehensively evaluated several major multi-class diagnostic models using gene expression data obtained from gene chips, and the results showed that multi-class support vector machines were the most effective classifiers [44].

In this study, we screened DRGs-lncRNAs prognostic prediction models associated with prognosis using the GEO database and COX proportional hazards regression analysis. We used the training set data to optimize the prediction model, and the test set data was used to verify the prediction effect of the model internally. We further performed statistical analysis on the function of the prognostic genes in the DRGs-lncRNAs prediction model and used GO and KEGG of the clusterProfiler package to enrich these significantly related genes, respectively. Subsequently, we validated the predictive model with tumor cell lines. In the future, if predictive models are to be applied to clinical practice, it is necessary to further validate the model in many clinical samples.

This study has some limitations. Firstly, the data source of this study was single, and the amount of data included was not large, so the analysis results may have some bias. Secondly, in order to ensure the correctness of the prognosis model, we needed to further confirm the prognosis model in other independent cohorts to ensure its accuracy. Thirdly, functional experiments should be performed to further elucidate the underlying molecular mechanisms for predicting the effects of DRG-lncRNAs.

## CONCLUSIONS

In conclusion, this study established a novel model of DRG-lncRNAs that can independently predict the OS of LUAD patients. Through analysis of immune cell infiltration and drug sensitivity, it was demonstrated that DRG-lncRNAs markers are closely related to immune cell infiltration and chemotherapy drug sensitivity. The DRG-lncRNAs risk prediction model constructed in this study can serve as an important method for predicting whether LUAD patients can benefit from immunotherapy and chemotherapy.

## Supplementary Materials

Supplementary Tables
