# Supplementary material for: Disulfidptosis-related lncRNAs signature predicting prognosis and immunotherapy effect in lung adenocarcinoma
Source: Aging (Albany NY). 2024 Jun 10;16(11):9972–89. doi: 10.18632/aging.205911 (PMC11210254; doi:10.18632/aging.205911)
Supplement: Supplementary Tables [file aging-16-205911-s001.pdf]

SUPPLEMENTARY TABLES

Supplementary Table 1. The sequence of primers of DRG-lncRNAs.

Gene		Primer (5′–3′)
**AL365181.2**	Forward	ATGGTTAGAGAATGGGAGAGGAG
	Reverse	AGAGTTGGCTTCGGAGGAAAT
**AC090559.1**	Forward	TCAGCGACGGAAAGAGTATGA
	Reverse	CCACTGGTTTCTGACTGGATGT
**AC090559.1**	Forward	CAAGTACATTGCTCCTCCTGAG
	Reverse	ACATCTGCTGGAAGGTGGACA
**GSEC**	Forward	TCGAAATGGACCCCAACTA
	Reverse	TGCACTTCTCCGACGTCC
**AC090559.1**	Forward	TGTGCCATACCATTAAACAGG
	Reverse	GCCTCT GATTGAAAATGAGAAC
**GAPDH**	Forward	GAACGGGAAGCTCACTGG
	Reverse	GCCTGCTTCACCACCTTCT

Supplementary Table 2. The expression of DRG-lncRNAs in LUAD.

Disulfidptosis	lncRNA	cor	*p*-value	Regulation
LRPPRC	LYRM4-AS1	0.4616865	6.47E-30	positive
SLC7A11	AL365181.3	0.48665534	1.63E-33	positive
NDUFA11	AC010331.1	0.40766643	4.46E-23	positive
NDUFA11	AC011498.6	0.42719621	2.08E-25	positive
NDUFA11	AC092171.4	0.4201018	1.52E-24	positive
LRPPRC	EMSLR	0.40842408	3.64E-23	positive
NDUFA11	SNHG10	0.41122497	1.72E-23	positive
SLC7A11	LINC01269	0.43523783	2.05E-26	positive
NDUFA11	AC027601.1	0.48489064	2.99E-33	positive
NDUFA11	AL390719.2	0.4646525	2.50E-30	positive
TLN1	AP001189.3	0.48633963	1.82E-33	positive
NDUFA11	AC008764.6	0.49134708	3.18E-34	positive
LRPPRC	AC091057.1	0.43879128	7.24E-27	positive
NDUFA11	AP006621.4	0.40495252	9.14E-23	positive
NDUFA11	AC008608.2	0.45525499	4.91E-29	positive
NDUFS1	MIR4713HG	0.40761158	4.52E-23	positive
RPN1	STK4-AS1	0.42887143	1.29E-25	positive
NCKAP1	STARD7-AS1	0.40501191	9.00E-23	positive
NDUFA11	AC087741.1	0.4119552	1.41E-23	positive
NDUFS1	AC015922.2	0.41801481	2.71E-24	positive
NCKAP1	AC015922.2	0.40355983	1.32E-22	positive
NDUFA11	ARRDC1-AS1	0.46392801	3.16E-30	positive
NDUFA11	AC116407.2	0.4070504	5.25E-23	positive
NDUFA11	PP7080	0.42846866	1.45E-25	positive
NDUFA11	AL033527.2	0.43450003	2.55E-26	positive
NDUFA11	AC084125.2	0.42219904	8.49E-25	positive
NDUFA11	AC015912.3	0.44201762	2.78E-27	positive
NCKAP1	AL606834.1	0.41467274	6.76E-24	positive
LRPPRC	RNASEH1-AS1	0.43314533	3.77E-26	positive
NDUFA11	C9orf163	0.4539165	7.44E-29	positive
NDUFA11	AC020558.2	0.40452698	1.02E-22	positive
PRDX1	AL139039.3	0.43287069	4.09E-26	positive

NCKAP1	AC112220.2	0.42661453	2.45E-25	positive
FLNB	AC112220.2	0.4288204	1.31E-25	positive
LRPPRC	KTN1-AS1	0.47778777	3.34E-32	positive
NDUFA11	AC114730.3	0.41930062	1.90E-24	positive
PRDX1	AC145207.8	0.41401517	8.09E-24	positive
NDUFA11	AC016773.1	0.450647	2.04E-28	positive
FLNB	MANCR	0.40514394	8.69E-23	positive
NDUFS1	OIP5-AS1	0.45065684	2.04E-28	positive
NCKAP1	OIP5-AS1	0.45918296	1.43E-29	positive
NDUFA11	AC087289.2	0.48790871	1.06E-33	positive
NDUFA11	AC011462.4	0.41035127	2.18E-23	positive
NDUFA11	CACTIN-AS1	0.43957162	5.75E-27	positive
NDUFA11	AC133552.5	0.40334438	1.39E-22	positive
NCKAP1	FGD5-AS1	0.4255597	3.30E-25	positive
NDUFA11	AC009065.4	0.50823316	7.14E-37	positive
NDUFA11	AC132872.2	0.46745917	1.01E-30	positive
SLC7A11	AL365181.2	0.41424494	7.60E-24	positive
NCKAP1	AL606489.1	0.42363616	5.68E-25	positive
OXSM	GAS5	0.46299476	4.26E-30	positive
NDUFA11	MHENCR	0.47477316	9.16E-32	positive
NDUFA11	AP001412.1	0.45967052	1.23E-29	positive
NUBPL	AC011815.1	0.48655792	1.69E-33	positive
NDUFA11	KMT2E-AS1	0.55066054	3.21E-44	positive
NDUFA11	AL928654.2	0.41029518	2.21E-23	positive
NUBPL	AL132800.1	0.47590879	6.27E-32	positive
ACTB	AC093673.1	0.51863033	1.41E-38	positive
NDUFA11	SNHG12	0.49235071	2.23E-34	positive
TLN1	PTPRN2-AS1	0.41229731	1.29E-23	positive
NDUFA11	PTOV1-AS2	0.50913148	5.12E-37	positive
NDUFA11	AL513320.1	0.475216	7.90E-32	positive
NUBPL	AC004943.2	0.41759744	3.04E-24	positive
SLC7A11	LINP1	0.44014239	4.86E-27	positive
NDUFA11	SNHG11	0.40738953	4.80E-23	positive
LRPPRC	AC234917.3	0.45836847	1.85E-29	positive
OXSM	ENTPD3-AS1	0.46439818	2.72E-30	positive
RPN1	AC137695.3	0.49766526	3.38E-35	positive
TLN1	AC006033.2	0.40671214	5.74E-23	positive
SLC3A2	AP003119.2	0.41510433	6.01E-24	positive
PRDX1	AC026785.3	0.46229842	5.32E-30	positive
TLN1	AL450326.1	0.44880561	3.59E-28	positive
NDUFA11	AC074212.1	0.43588729	1.70E-26	positive
NDUFA11	MIR762HG	0.41873006	2.22E-24	positive
NDUFA11	LMNTD2-AS1	0.41173916	1.50E-23	positive
NDUFA11	NALT1	0.42555416	3.31E-25	positive
NCKAP1	NORAD	0.5041224	3.25E-36	positive
NDUFA11	CAHM	0.44866595	3.74E-28	positive
LRPPRC	AC003086.1	0.42178669	9.52E-25	positive
PRDX1	AC092115.3	0.42534814	3.51E-25	positive
PRDX1	AC106045.1	0.41648121	4.13E-24	positive
PRDX1	GSEC	0.44076205	4.04E-27	positive
NDUFA11	LINC01023	0.42020526	1.48E-24	positive
NDUFA11	AC005387.1	0.53310944	4.71E-41	positive
RPN1	AC104472.4	0.41285638	1.11E-23	positive
NCKAP1	AC021037.1	0.44649135	7.25E-28	positive
NDUFA11	AL355353.1	0.40890105	3.21E-23	positive
ACTB	C10orf55	0.43251269	4.53E-26	positive

NDUFA11	AP003419.3	0.40444853	1.04E-22	positive
NDUFA11	LINC01089	0.48277	6.19E-33	positive
FLNA	AL645939.5	0.4022659	1.85E-22	positive
LRPPRC	AC012073.1	0.51295597	1.22E-37	positive
LRPPRC	SNHG16	0.53660407	1.14E-41	positive
NDUFA11	AC008760.1	0.4164544	4.16E-24	positive
NDUFA11	LINC01311	0.4642363	2.86E-30	positive
RPN1	LINC01063	0.44878181	3.61E-28	positive
TLN1	AL513165.1	0.40699338	5.33E-23	positive
LRPPRC	ALMS1-IT1	0.41196351	1.41E-23	positive
NUBPL	AL139353.2	0.45900853	1.51E-29	positive
NDUFS1	AL590666.2	0.40146436	2.28E-22	positive
SLC7A11	AL590666.2	0.44747427	5.38E-28	positive
GYS1	LINC01960	0.40149113	2.26E-22	positive
NCKAP1	USP46-DT	0.44039088	4.51E-27	positive
SLC3A2	AC092718.4	0.42561952	3.25E-25	positive
TLN1	AC090559.1	0.46384241	3.25E-30	positive
NDUFA11	AC012615.1	0.52549242	9.77E-40	positive
NDUFA11	TMEM147-AS1	0.40948932	2.74E-23	positive
NDUFA11	AL136295.2	0.43484344	2.30E-26	positive
